# Supplementary material for: An agent-based model on antimicrobial de-escalation in intensive care units: Implications on clinical trial design
Source: PLoS One. 2024 Apr 16;19(4):e0301944. doi: 10.1371/journal.pone.0301944 (PMC11020418; doi:10.1371/journal.pone.0301944)
Supplement: S1 Appendix — (PDF) [file pone.0301944.s001.pdf]

An Agent-Based Model on Antimicrobial De-escalation in Intensive Care Units: Implications on Clinical Trial Design
Supplementary Information

Xi Huo[†*1] and Ping Liu[†2]

[1]Department of Mathematics, University of Miami, Coral Gables, Florida, USA
[2]Department of Computer Science, Illinois Institute of Technology, Chicago, Illinois, USA

Contents

1 Baseline Setup **1**

2 Status Change Scenarios **2**
2.1 Contact between HCW and each patient . . . 2
2.2 Infection development . . . 7
2.3 Drug change . . . 7
2.3.1 Model 1: De-escalation Group . . . 7
2.3.2 Model 2: Control Group . . . 7
2.4 Completion of treatment . . . 8
2.5 Discharge and admission . . . 8
2.6 Deaths . . . 8
2.7 HCW status . . . 8

3 Intrinsic Mutation **9**

4 Simulation Outputs **10**

5 Outcome Measurements **10**

1 Baseline Setup

1. Denote CIP by drug A, TZP by drug B, last-resort drugs by drug C, non-PA de-escalated drugs by drug L.

2. Denote susceptible PA strain by strain 0, CIP-resistant PA strain by strain 1, TZP-resistant PA strain by strain 2, Dual-resistant strain by strain 12, non-PA species by species X.

3. Main setup: $4n$ patients, n HCWs, 5 pathogens $(0, 1, 2, 12, X)$, 4 drugs (A, B, C, L). HCW visits each patient routinely, and spends two hours every visit.

4. Patient attributes: **infection status, drug use, treatment time, conversion time, dominant strain, lab result, time in ICU, super-infection, and infection time**.

5. HCW attributes: **contaminated strains**.

*Correspondence: x.huo@math.miami.edu
†Contributed equally to this work.

Status	Values	Remark	Upon Admission
	Patient status		
**infection status**	uninfected, infected		uninfected
**drug use**	A, B, C, L or Null		Null
**treatment time**	≥ 0 or Null	increases with time	Null
**conversion time**	≥ 0 or Null	not increase with time	Null
**infection time**	$[0, 10]$ or Null	not increase with time	see section 2.2 on infection development
**dominant strain**	$0, 1, 2, 12, X_A, X_N$		X_N: $1 - m$;<br>X_A: $(1 - a) \cdot m$.<br>0: $a \cdot m \cdot (1 - r_1 - r_2)$;<br>1: $a \cdot m \cdot r_1$;<br>2: $a \cdot m \cdot r_2$;
**lab result**	$0, 1, 2, 12, X$ or Null		Null
**time in ICU**	≥ 0	increases with time	0
**super-infection**	N or Y		N
	HCW status		
**contaminated strains**	subset of $\{0, 1, 2, 12\}$		Null

Table 0: Attribute Values. X_N refers to patients colonized by non-PA species without prior exposure to PA antibiotics, and X_A refers to those who have recent exposure to PA antibiotics.

2 Status Change Scenarios

In the following, tables labeled by "v1" refers to the de-escalation model (Model 1), and tables labeled by "v2" refers to the control group model (Model 2).

2.1 Contact between HCW and each patient

**HCW status before contact**	**Probability: patient status after contact**
HCW does not carry bacteria	• 1: nothing changes.
HCW carries one bacteria strain i	• $1 - p$: nothing changes.<br>• p: dominant strain $= i$.
HCW carries two bacteria strains i and j	• $1 - p$: nothing changes.<br>• p: dominant strain $= i$ or j with equal probability 1/2.
HCW carries three bacteria strains i, j and k	• $1 - p$: nothing changes.<br>• p: dominant strain $= i$, j, or k with equal probability 1/3.
HCW carries all four bacteria strains	• $1 - p$: nothing changes.<br>• p: dominant strain $= 0, 1, 2$, or 12 with equal probability 1/4.

Table 1: Change of Patient Status Upon Contact. For patient infection status = uninfected and dominant strain $= X_A$ before contact.

**HCW status before contact**	**Probability: HCW status after contact**
HCW does not carry patient's bacteria	• $1 - q$: nothing changes.<br>• q: include strain i (patient's dominant strain) in HCW's strains.
HCW carries the same strain as patient's	• 1: nothing changes.

Table 2: Change of HCW Status Upon Contact. For patient infection status = uninfected and dominant strain contains $0, 1, 2$ or 12 before contact. If dominant strain contains only X then nothing happens to HCW.

<table>
<tr><th>Patient status
before contact</th><th>HCW status before contact</th><th>All status after contact</th></tr>
<tr><td rowspan="4">dominant strain = 0 or 1</td><td>HCW does not carry 2 or 12</td><td>• no change for patient status.</td></tr>
<tr><td>HCW carries 2</td><td>• r: patient's dominant strain = 2
IF dominant strain= 1:
set conversion time = current time − treatment time + 3;
IF dominant strain= 0: do not set conversion time.
• $1 - r$: no change for patient's status.</td></tr>
<tr><td>HCW carries 12</td><td>• r: patient's dominant strain = 12
AND set conversion time = current time − treatment time + 3;
• $1 - r$: no change for patient's status.</td></tr>
<tr><td>HCW carries both 2 and 12</td><td>• r: patient's dominant strain = 2 or 12 with equal probability 1/2;
if switch to strain 2:
IF dominant strain= 1:
set conversion time = current time − treatment time + 3;
IF dominant strain= 0: do not set conversion time.
if switch to strain = 12 set:
conversion time = current time − treatment time + 3;
• $1 - r$: no change for patient's status;</td></tr>
<tr><td>dominant strain= 2 or 12</td><td>Any situation</td><td>• no change for patient status.</td></tr>
<tr><td colspan="3">In the above cases, include patient's before-contact-dominant-strain in HCW's strains with a probability of q.</td></tr>
<tr><td colspan="3">In the cases below, no change for HCW status after each contact.</td></tr>
<tr><td rowspan="4">dominant strain = X</td><td>HCW does not carry 2 or 12</td><td>• no change for patient status.</td></tr>
<tr><td>HCW carries 2</td><td>• r: patient's dominant strain = 2;
AND set super-infection=Y
AND set conversion time = current time − treatment time + 3;
• $1 - r$: no change for patient status.</td></tr>
<tr><td>HCW carries 12</td><td>• r: patient's dominant strain = 12;
AND set super-infection=Y
AND set conversion time = current time − treatment time + 3;
• $1 - r$: no change for patient status.</td></tr>
<tr><td>HCW carries both 2 and 12</td><td>• r: patient's dominant strain = 2 or 12 with equal probability 1/2;
AND set super-infection=Y
AND set conversion time = current time − treatment time + 3;
• $1 - r$: no change for patient status.</td></tr>
</table>

Table 3.1.v1: Change of Status Upon Contact. For patient infection status = infected before contact and treatment time $\in [0, 3]$.

<table>
<tr><th>Patient status
before contact</th><th>HCW status before contact</th><th>All status after contact</th></tr>
<tr><td rowspan="4">dominant strain = 0 or 1</td><td>HCW does not carry 2 or 12</td><td>• no change for patient status.</td></tr>
<tr><td>HCW carries 2</td><td>• r: patient's dominant strain = 2
set conversion time = current time − treatment time + 3.
• $1 - r$: no change for patient's status.</td></tr>
<tr><td>HCW carries 12</td><td>• r: patient's dominant strain = 12
AND set conversion time = current time − treatment time + 3;
• $1 - r$: no change for patient's status.</td></tr>
<tr><td>HCW carries both 2 and 12</td><td>• r: patient's dominant strain = 2 or 12 with equal probability 1/2;
conversion time = current time − treatment time + 3;
• $1 - r$: no change for patient's status;</td></tr>
<tr><td>dominant strain= 2 or 12</td><td>Any situation</td><td>• no change for patient status.</td></tr>
<tr><td colspan="3">In the above cases, include patient's before-contact-dominant-strain in HCW's strains with a probability of q.</td></tr>
<tr><td colspan="3">In the cases below, no change for HCW status after each contact.</td></tr>
<tr><td rowspan="4">dominant strain = X</td><td>HCW does not carry 2 or 12</td><td>• no change for patient status.</td></tr>
<tr><td>HCW carries 2</td><td>• r: patient's dominant strain = 2;
AND set super-infection=Y
AND set conversion time = current time − treatment time + 3;
• $1 - r$: no change for patient status.</td></tr>
<tr><td>HCW carries 12</td><td>• r: patient's dominant strain = 12;
AND set super-infection=Y
AND set conversion time = current time − treatment time + 3;
• $1 - r$: no change for patient status.</td></tr>
<tr><td>HCW carries both 2 and 12</td><td>• r: patient's dominant strain = 2 or 12 with equal probability 1/2;
AND set super-infection=Y
AND set conversion time = current time − treatment time + 3;
• $1 - r$: no change for patient status.</td></tr>
</table>

Table 3.1.v2: Change of Status Upon Contact. For patient infection status = infected before contact and treatment time $\in [0, 3]$.

**Patient status before contact**	**HCW status before contact**	**All status after contact**
drug use = A and dominant strain= 0 or 2	HCW carries either 1 or 12	• r: patient's dominant strain=1 or 12<br>AND set conversion time = current time;<br>• $1-r$: no change for patient status.
	HCW carries both 1 and 12	• r: patient's dominant strain = 1 or 12 with equal probability 1/2<br>AND set conversion time = current time;<br>• $1-r$: no change for patient status.
	HCW carries neither 1 or 12	• no change for patient status.
drug use = A and dominant strain= 1 or 12	HCW with all possible status	• no change for patient status.
drug use = B and dominant strain= 2 or 12	HCW with all possible status	• no change for patient status.
drug use = B and dominant strain= 0 or 1	HCW carries either 2 or 12	• r: patient's dominant strain=2 or 12<br>AND set conversion time = current time;<br>• $1-r$: no change for patient status.
	HCW carries both 2 and 12	• r: patient's dominant strain = 2 or 12 with equal probability 1/2<br>AND set conversion time = current time;<br>• $1-r$: no change for patient status.
	HCW carries neither 2 or 12	• no change for patient status.
drug use = C	HCW with all possible status	• no change for patient status.
drug use = L and dominant strain $\neq X$	HCW with all possible status	• no change for patient status.
**In the above cases, include patient's before-contact-dominant-strain in HCW's strains with a probability of q.**		
**In the cases below, no change for HCW status after each contact.**		
drug use = L and dominant strain= X	HCW carries one strain i	• s: patient's dominant strain=i<br>AND set conversion time = current time<br>AND set super-infection = Y;<br>• $1-s$: no change for patient status.
	HCW carries two i and j	• s: patient's dominant strain = i or j with equal probability 1/2;<br>AND set conversion time = current time<br>AND set super-infection=Y;<br>• $1-s$: no change for patient status.
	HCW carries i, j and k	• s: patient's dominant strain = i or j or k with equal probability 1/3;<br>AND set conversion time = current time<br>AND set super-infection=Y;<br>• $1-s$: no change for patient status.
	HCW carries all four strains	s: patient's dominant strain = 0 or 1 or 2 or 12 with equal probability 1/4;<br>AND set conversion time = current time<br>AND set super-infection=Y;<br>• $1-s$: no change patient status.

Table 3.2.v1: Change of Status Upon Contact. For patient infection status = infected before contact and treatment time > 3.

**Patient status before contact**	**HCW status before contact**	**All status after contact**
drug use = A and dominant strain= 0 or 2	HCW carries either 1 or 12	• r: patient's dominant strain=1 or 12<br>AND set conversion time = current time;<br>• $1-r$: no change for patient status.
	HCW carries both 1 and 12	• r: patient's dominant strain = 1 or 12 with equal probability 1/2<br>AND set conversion time = current time;<br>• $1-r$: no change for patient status.
	HCW carries neither 1 or 12	• no change for patient status.
drug use = A and dominant strain= 1 or 12	HCW with all possible status	• no change for patient status.
drug use = B and dominant strain= 2 or 12	HCW with all possible status	• no change for patient status.
drug use = B and dominant strain= 0 or 1	HCW carries either 2 or 12	• r: patient's dominant strain=2 or 12<br>AND set conversion time = current time;<br>• $1-r$: no change for patient status.
	HCW carries both 2 and 12	• r: patient's dominant strain = 2 or 12 with equal probability 1/2<br>AND set conversion time = current time;<br>• $1-r$: no change for patient status.
	HCW carries neither 2 or 12	• no change for patient status.
drug use = C	HCW with all possible status	• no change for patient status.
**In the above scenarios, include patient's before-contact-dominant-strain in HCW's strains with a probability of q.**		
**In the below scenarios, do not include patient's before-contact-dominant-strain in HCW's strains.**		
drug use = B and dominant strain= X	HCW does not carry 2 or 12	• no change for patient status.
	HCW carries 2	• r: patient's dominant strain = 2;<br>AND set super-infection=Y<br>AND set conversion time = current time;<br>• $1-r$: no change for patient status.
	HCW carries 12	• r: patient's dominant strain = 12;<br>AND set super-infection=Y<br>AND set conversion time = current time;<br>• $1-r$: no change for patient status.
	HCW carries both 2 and 12	• r: patient's dominant strain = 2 or 12 with equal probability 1/2;<br>AND set super-infection=Y<br>AND set conversion time = current time;<br>• $1-r$: no change for patient status.

Table 3.2.v2: Change of Status Upon Contact. For patient infection status = infected before contact and treatment time > 3.

2.2 Infection development

Upon every patient's admission, the patient is assigned with an **infection time**:

- if **dominant strain**= X_A or X_N:
  - first determine if this patient will develop infection at a probability of σ_x, then assign the **infection time** in between 0 to 5 days with a uniform distribution;
  - upon any strain change from X to others, check if the patient's **infection status**=uninfected:
    - if yes, determine if the patient will develop infection at a probability of σ_c:
      - if yes, draw a number NN from 0 to 5 with uniform distribution and replace the **infection time** by **ICU time**+NN;
      - if no, replace the **infection time** by NULL
    - if no, do nothing.
- if **dominant strain**= $0, 1, 2, 12$ first determine if this patient will develop infection at a probability of σ_c, then assign the **infection time** in between 0 to 5 days with uniform distribution.

when **infection time=ICU time**, an infection development happens, set:

- **infection status** changes from *uninfected* to *infected*;
- **drug use** = B;
- **treatment time** = 0;
- **lab result** = **dominant strain** if **dominant strain**= $0, 1, 2, 12$;
  lab result = X if **dominant strain**= X_A or X_N.

2.3 Drug change

2.3.1 Model 1: De-escalation Group

The **drug use** status is changed only when:

1. **infection status** changes from *uninfected* to *infected*: set **drug use** = B.
2. **treatment time** reaches 3 (days): then set
   - **drug use** = A if **lab result** = 0 or 2;
   - **drug use** = B if **lab result** = 1;
   - **drug use** = C if **lab result** = 12;
   - **drug use** = L if **lab result** = X.
3. current time $-$ **conversion time** = 3 (days): if **drug use** = A or B - now set **drug use** = C;
   if **drug use** = L - then do the followings:
   - set **drug use** = B;
   - reset **treatment time** = 0;
   - reset **lab result** = **dominant strain**.

2.3.2 Model 2: Control Group

The **drug use** status is changed only when:

1. **infection status** changes from *uninfected* to *infected*: set **drug use** = B.
2. **treatment time** reaches 3 (days): then set
   - **drug use** = A if **lab result** = 2;
   - **drug use** = B if **lab result** = $0, 1$ or X;
   - **drug use** = C if **lab result** = 12.
3. current time $-$ **conversion time** = 3 (days):
   then **drug use** = A or B - so set **drug use** = C.

2.4 Completion of treatment

Treatment is terminated if:

1. **conversion time** = Null and **lab result** $= 0, 1$ and **treatment time** = 7 (days): then set
   - **infection status** = *uninfected*;
   - **treatment time** = **conversion time** = **lab result** = **drug use** = Null.
2. **conversion time** = Null and **lab result** $= 2, 12$ and **treatment time** = 10 (days): then set
   - **infection status** = *uninfected*;
   - **treatment time** = **conversion time** = **lab result** = **drug use** = Null.
3. **conversion time** $\neq$ Null and **current time** $-$ **conversion time** = 10 (days): then set
   - **infection status** = *uninfected*;
   - **treatment time** = **conversion time** = **lab result** = **drug use** = Null.

2.5 Discharge and admission

Discharge happens as a probability function that depends on the patient's ICU time

- μ(ICU time) for those who has **infection status** = *uninfected* and has **time in ICU** ≥ 2 (days), where the value of the probability is read from file dischargeprob;
- μ^- for those who has **infection status** = *infected* and has **time in ICU** ≥ 2 (days), where the value of the probability is $\mu(\text{ICU time}) \cdot \kappa_\mu$ with κ_μ being the hazard ratio of discharge for infected patients.

If any patient is discharged, reset all status to default.

2.6 Deaths

Death occurs at a probability function that depends on the patient's ICU time:

- ν(ICU time) for patients with **conversion time**=Null or current time $-$ conversion time > 3, where the value of the probability is read from file deathprob;
- ν^+(ICU time) for patients with **conversion time**$\neq$ Null and current time $-$ conversion time < 3 days, where the value of the probability is $\nu(\text{ICU time}) \cdot \kappa_\nu^+$, where κ_ν^+ is the hazard ratio of death for patients with inappropriate antimicrobials.

If death happens, reset all status to default.

2.7 HCW status

Status of HCW will only change if

1. a contact happens (see contact rules);
2. set all bacteria strains of HCW to be empty every 8 hours;
3. set all bacteria strains of HCW to be empty at a probability of η after each contact.

3 Intrinsic Mutation

Intrinsic mutation could happen at a probability of $\varepsilon = 0.01$ to any patient, and is determined only once per day based on the following rules.

Patient status	Probability: status update
dominant strain = 0	• ε: patient's dominant strain = 2; do not set conversion time. • $1 - \varepsilon$: nothing changes.
dominant strain = 1	• ε: patient's dominant strain = 12; set conversion time = current time − treatment time +3. • $1 - \varepsilon$: nothing changes.

Table 4.1.v1: Change of Patient Status Upon Mutation. For patient infection status = *infected* and treatment time $\in [0, 3]$.

Patient status	Probability: status update
dominant strain = 0	• ε: patient's dominant strain = 2; set conversion time = current time − treatment time +3. • $1 - \varepsilon$: nothing changes.
dominant strain = 1	• ε: patient's dominant strain = 12; set conversion time = current time − treatment time +3. • $1 - \varepsilon$: nothing changes.

Table 4.1.v2: Change of Patient Status Upon Mutation. For patient infection status = *infected* and treatment time $\in [0, 3]$.

Patient status	Probability: status update
drug use = A and dominant strain = 0	• ε: patient's dominant strain = 1; set conversion time = current time. • $1 - \varepsilon$: nothing changes.
drug use = A and dominant strain = 2	• ε: patient's dominant strain = 12; set conversion time = current time. • $1 - \varepsilon$: nothing changes.
drug use = B and dominant strain = 0	• ε: patient's dominant strain = 2; set conversion time = current time. • $1 - \varepsilon$: nothing changes.
drug use = B and dominant strain = 1	• ε: patient's dominant strain = 12; set conversion time = current time. • $1 - \varepsilon$: nothing changes.

Table 4.2.v1: Change of Patient Status Upon Mutation. For patient infection status = *infected* and treatment time > 3.

**Patient status**	**Probability: status update**
drug use = A and dominant strain = 0	• ε: patient's dominant strain = 1; set conversion time = current time. • $1-\varepsilon$: nothing changes.
drug use = A and dominant strain = 2	• ε: patient's dominant strain = 12; set conversion time = current time. • $1-\varepsilon$: nothing changes.
drug use = B and dominant strain = 0	• ε: patient's dominant strain = 2; set conversion time = current time. • $1-\varepsilon$: nothing changes.
drug use = B and dominant strain = 1	• ε: patient's dominant strain = 12; set conversion time = current time. • $1-\varepsilon$: nothing changes.

Table 4.2.v2: Change of Patient Status Upon Mutation. For patient infection status = *infected* and treatment time > 3.

4 Simulation Outputs

Measurements in the following table will be updated during every time step (2 hrs), but their values are stored only for EACH DAY:

**Measurement**	**Increment**	**Decrement**
Admission	a discharge or death event happens	N/A
Death	a death event happens	N/A
Discharge	a discharge event happens	N/A
Infection-i (i=X,0,1,2,12)	infection development with dominant strain= i; or strain conversion to i occurs.	completion of treatment or death occurs to an infected patient with dominant strain= i; or a strain conversion from i occurs.
LabResult-i ($i = 0, 1, 2, 12$)	a patient's lab result is switched from Null to i	N/A
Superinfection	a patient's super-infection status switch from N to Y	completion of treatment occurs to patient with super-infection status Y
CumSuperinfection	a patient's super-infection status switch from N to Y	N/A
Colonization-i ($i = 0, 1, 2, 12$)	any event in Table 1 happens to a patient ending with a switch to dominant strain= i	N/A
MisEmpiric	a strain conversion in Table 3.1 with conversion time being set	N/A
TempEmpiric	a strain conversion in Table 3.1 with conversion time NOT being set	N/A
DrugUseDef-i ($i = A, B, C, L$)	a drug change to i happens in 2.3.v.2	N/A
DrugUseCorr-i ($i = A, B, C, L$)	a drug change to i happens in 2.3.v.3	N/A
Transmission-i ($i = 0, 1, 2, 12$)	a transmission happens in Table 3.1 or 3.2	N/A
Mutation-i ($i = 1, 2, 12$)	a mutation happens in Table 4.1 or 4.2	N/A

Table 5: Simulation Outputs

5 Outcome Measurements

For each experiment, we evaluate the following outcome measurements by the end of every week.

- **CIP-resistant infections** - incidence rate of CIP-resistant strain infections, measured as the number of CIP-resistant strain infection incidences in 100 admitted patients.
  $100 \cdot$ (LabResult-1+Transmission-1+Mutation-1)/Admission.
- **TZP-resistant infections** - incidence rate of TZP-resistant strain infections, measured as the number of TZP-resistant strain infection incidences in 100 admitted patients.
  $100 \cdot$ (LabResult-2+Transmission-2+Mutation-2)/Admission.
- **Dual-resistant infections** - incidence rate of dual-resistant strain infections, measured as the number of dual-resistant strain infection incidences in 100 admitted patients.
  $100 \cdot$ (LabResult-12+Transmission-12+Mutation-12)/Admission.
- **Cumulative PA infections** - incidence rate of *P. aeruginosa* infections, measured as the number of *P. aeruginosa* infection incidences in 100 admitted patients.
  $100 \cdot \sum_{i=0,1,2,12}$(LabResult-i+Transmission-i+Mutation-i)/Admission.
- **CIP-resistant strain colonization** - incidence rate of CIP-resistant strain colonization, measured as the number of CIP-resistant strain colonization incidences in 100 admitted patients.
  $100 \cdot$ (Colonization-1)/Admission.
- **TZP-resistant strain colonization** - incidence rate of TZP-resistant strain colonization, measured as the number of TZP-resistant strain colonization incidences in 100 admitted patients.
  $100 \cdot$ (Colonization-2)/Admission.
- **Dual-resistant strain colonization** - incidence rate of dual-resistant strain colonization, measured as the number of dual-resistant strain colonization incidences in 100 admitted patients.
  $100 \cdot$ (Colonization-12)/Admission.
- **Mutations against CIP** - incidence rate of CIP-resistance mutation development, measured by the number of CIP-resistant mutation developments in 100 admitted patients.
  $100 \cdot$ (Mutation-1)/Admission.
- **Mutations against TZP** - incidence rate of TZP-resistance mutation development, measured by the number of TZP-resistant mutation developments in 100 admitted patients.
  $100 \cdot$ (Mutation-2)/Admission.
- **Mutations against CIP& TZP** - incidence rate of dual-resistance mutation development, measured by the number of dual-resistant mutation developments in 100 admitted patients.
  $100 \cdot$ (Mutation-12)/Admission.
- **Use of TZP** - use of TZP, measured by the number of patients who receive TZP in their definitive or correction therapies in 100 admitted patients.
  $100 \cdot$ (DrugUseDef-B+DrugUseCorr-B)/Admission.
- **Use of last-resort drugs** - use of drugs for dual-resistant PA infections, measured by the number of patients who receive the last-resort drug in 100 admitted patients.
  $100 \cdot$ (DrugUseDef-C+DrugUseCorr-C)/Admission.
- **Ineffective empiric treatments** - ineffective empiric treatments, measured as the number of patients who receive ineffective antibiotics during empiric therapies in 100 admitted patients.
  $100 \cdot$ (MisEmpiric+LabResult-2+LabResult-12)/Admission.
- **Cumulative super-infections** - incidence rate of super-infection, measured as the number super-infected patients in 100 admitted patients.
  $100 \cdot$ (CumSuperinfection)/Admission.
- **Deaths** - probability of death, measured as the percentage of patients who die in the ICU.
  $100 \cdot$ (Death)/Admission.
