# Supplementary material for: An agent-based model on antimicrobial de-escalation in intensive care units: Implications on clinical trial design
Source: PLoS One. 2024 Apr 16;19(4):e0301944. doi: 10.1371/journal.pone.0301944 (PMC11020418; doi:10.1371/journal.pone.0301944)
Supplement: Attachment — Submitted filename: Response to Reviewers_HuoLiu.pdf [file pone.0301944.s002.pdf]

Response to Reviewers

***__Reviewer #1__*:** *In this manuscript, the authors use an agent based model to predict the effects of antibiotic de-escalation (a treatment regime that switches from a broad-spectrum empiric drug to a more specific drug following susceptibility testing). The authors present qualitative results on the effects of de-escalation on colonisation/infection by different strains and quantitative results on the sample size required to detect effects in clinical trials.*

The paper is generally clear and the idea of using ABMs to estimate sample sizes for clinical trials is in principle very interesting. Furthermore, fitting to data to estimate transmission parameters is welcome. However, in the presence of considerable uncertainty around other parameters and model structure, I did not think all of the conclusions (in particular quantitative ones) are altogether justified by the analysis.

Firstly, I have quite a lot of queries about the model structure and in particular the ecological interactions in the model. My background is in antibiotic resistance modelling, but not of pseudomonas specifically. Many of the features of the model are very different from what I am used to seeing. For example:

-All patients are either colonised or infected. Antibiotic treatment leads to infection being resolved, but a reversion to the colonised state (i.e. colonisation is not affected by antibiotics).

Response: Colonized patients do not have symptoms of infection, and only patients with bacterial infections are treated with antibiotics. Healthcare-associated infections (HAIs) include central line-associated bloodstream infections, catheter-associated urinary tract infections, and ventilator-associated pneumonia, which are caused by the transmission of pathogens from invasive devices and procedures to treat patients. Thus all patients are vulnerable to infections caused by bacteria in their bodies. Therefore, we assume uninfected patients are colonized by either *Pseudomonas* or other species and assume patients colonized by other species have a lower rate of infection development to reflect the lower possibility of infection.

We assume patients with resolved infections are still colonized because patients remain *Pseudomonas* carriers for 12-25 days (doi: 10.1016/S0369-8114(01)00219-X), which is longer than the normal stay in the ICU (doi: 10.1007/s00134-007-0619-x; doi: 10.1007/s00134-015-4016-6; doi: 10.1007/s00134-016-4301-z).

- Resistant and sensitive strains only differ in their susceptibility to antibiotics (i.e. no fitness cost of resistance).

Response: We assume that the chances for patients and HCWs to contract resistant and sensitive strains are the same. To reflect the fitness cost of resistance, one can easily change the value of strain transmission probability with a lower transmission probability for the resistant strains. We did not make such an assumption because it is hard for one to estimate the impact of the fitness cost of resistance on the transmission probability. Another parameter distinguishing sensitive and resistant strains is the infection development probability for people colonized with different strains. In this study, we assume this infection development probability to be the same for each

strain, and this can also be easily modified to reflect the difference when relevant data become available.

- *There seems to be an ecological interaction between pseudomonas and the other species in terms of competition for colonisation, but this is not explicitly stated or fully explained (I assume this was why previous antibiotic exposure is necessary for pseudomonas to colonise hosts carrying other species).*

Response: We assume previous antibiotic exposure will result in pseudomonas colonization based on the findings of a few references (doi: 10.1186/cc10026; doi: 10.1007/s00134-007-0671-6; doi: 10.1053/jhin.2002.1370).

The ecological interaction between pseudomonas and other species appears in the process of strain conversion – which happens under the antibiotic selective pressure. We have incorporated a new illustrative figure (Figure 1(b)) in the updated manuscript with additional texts to clarify this procedure.

- *Healthcare workers can be contaminated with all species, but only transmit pseudomonas.*

Response: This phenomenon is based on the selective pressure of antibiotics under all scenarios:

1. When HCWs contact infected patients under pseudomonal antibiotics, the patients are vulnerable to pseudomonal strains that are resistant to the antibiotics they are currently taking, so the HCWs only transmit some resistant *Pseudomonas* strains.
2. When HCWs contact infected patients under non-pseudomonal antibiotics, the patients are vulnerable to pseudomonas strains that cannot be treated by the drugs they are taking, so the HCWs would transmit pseudomonas strains to patients.
3. When HCWs contact patients colonized by pseudomonas strains, no transmission would occur as there is no selective pressure.
4. When HCWs contact patients colonized by non-pseudomonal bacteria and with prior pseudomonas antibiotic exposures, the patients are vulnerable to pseudomonas strains, thus HCWs would transmit pseudomonas.

We hope the newly added text and figure on the antibiotic selective pressure mechanism (Figure 1(b)) could help clarify such concerns.

Of course, these features being different to what I am familiar with does not mean they are not reasonable. However, it was unclear from the manuscript to what extent these features are assumptions by the authors vs based on known pseudomonas ecology. It seems to me that some of the results the authors present - in particular the results relating to the effect of de-escalation on the non-pseudomonas species - will be very sensitive to these assumptions. Therefore without further discussion of these model features and, where appropriate references to literature, it is very difficult to interpret the model results.

Response: We hope our above response, together with the references, will clarify most of the model assumptions raised by the reviewer. We have incorporated a few of the references above in the updated version.

Secondly, I did not think the conclusion of 100 ICUs followed for 100 weeks to detect effects of de-escalation presented in the abstract was entirely meaningful. As the authors discuss and demonstrate in their sensitivity analysis, this depends on features of the ICU. In addition, there is most likely additional sensitivity to model structure and parameters not fully explored in the sensitivity analysis - in particular the mutation rate, which, as the authors discuss, is not based empirical evidence and could therefore be off by orders of magnitude.

Response: We have revised our conclusions on the sample size and study period needed to detect the effects of de-escalation in the new submission. Further, we have included more experiments to show that the major effects of de-escalation do not differ in ICU size, transmission intensity, or perturbations of parameters.

In addition, we have included references on the estimation of mutation rate in the revised manuscript so that the actual order of magnitude for the mutation probability is chosen with credible sources and arguments. A detailed discussion on converting the mutation probability from real data can be found in one study of our collaborators from Toronto teaching hospitals (doi: 10.1016/j.epidem.2012.12.001). Moreover, we have incorporated more references on our choices of all other parameters where all of them are estimated based on results found in many medical studies.

Additional points which I think would improve the readability/accessibility of the manuscript.

- *Many of the ecological assumptions were implicit in the model description. I think adding a brief overview describing these would be very helpful to the reader. I found Figure 1 very helpful and think further addition could make the figure even better (e.g. is there a difference between the cured and colonised states?; differences in the behaviour of different strains/species).*

Response: We have significantly modified our figures to illustrate the background with more clarity. Further, we have incorporated more details on the selective pressure and strain conversion mechanism in the updated manuscript.

- *Fitting to real data was a strength of this manuscript. I found the description of this fitting a little light in terms of detail, and would have liked to see a more thorough discussion of the approach used here.*

Response: We make sure to include the parameter calibration part in the updated manuscript, and we have added a new figure (Figure 3) to show our calibration results visually.

- *In the introduction, the authors mention their previous work using ODEs to look at the ecological effect of de-escalation. I would have found a contextualisation of the results here in light of that previous work interesting.*

Response: Using ODEs and agent-based models would provide analyses of the same topic from different perspectives. For example, the results obtained by ODEs are more used to give predictions of the system status after a sufficiently long time period and the outbreak potential of

the nosocomial pathogens. In comparison, results obtained by agent-based models are more practical in explaining observations of clinical studies and provide insights into the sample size and study period. Therefore, we found that comparing the results between studies with different tools is not the primary purpose of this study. We thus chose not to discuss the comparison in the current version. However, we will incorporate a discussion on the meaning of different models if the reviewer believes it is essential.

Reviewer #2: *Huo & Liu present an agent-based model of infection with drug-susceptible or resistant bacteria, designed to mimick the intensive care setting with patient/HCW transmission of bacteria, emergence of drug resistance, and antimicrobial prescription strategies. Here, the strategy of interest is de-escalation and the outcome is the incidence of drug-resistant infections. The authors use their model to estimate the sample size (no. of participating ICUs) that would be required in a controlled clinical trial to detect an impact of de-escalation on drug resistance. This is, in my opinion, the most important message of the paper. Other messages are not unexpected (reducing global use of a drug reduces pressure and, in fine, drug resistance) but this paper has merit and importance by showing that very large sample sizes or extended followup periods would be required to detect the impact of de-escalation. Beyond the question of de-escalation, the model itself has potential to address many other pending questions regarding drug prescription strategies, as discussed by the authors; however I could not access the code to evaluate its useability.*

Overall the study question is important, the conclusions are well supported by the data and the paper reads well. I appreciate that the baseline parameters for the model are based on literature review. I have some comments listed below.

MAJOR COMMENTS

§2.1 Model assumptions. The authors choose 2 drugs (pipe-tazo and ciprofloxacin) to represent empirical and definitive therapy. From an ecological standpoint these drugs do not exhibit cross-resistance (resistance to one drug does not imply resistance to the other drug). This can be helpful to ease interpretation of results but it does not reflect well the situation of de-escalation in many settings where de-escalation imply narrowing the drug spectrum among drugs with cross-resistance (e.g. de-escalating from carbapenems to cephalosporins, where carbapanem resistance frequently imply cephalosporin resistance but not the other way around), rather than changing the drug's class (betalactam vs quinolons here). This peculiarity of the model should be discussed in more depth as using drugs without cross-resistance is closer to the setting of antibiotic mixing/cycling than to the question of de-escalation in my opinion. As a consequence of the author's choice of drugs with non-overlapping resistance spectrum, the emergence of resistance to definitive therapy (here, ciprofloxacin) is emphasized and considered a trade-off in the paper, while in many practical situations there would be no such tradeoff: de-escalating from carbapenems to cephalosporins does not imply any resistance trade-off because continuing carbapenem treatement would select for cephalosporin resistance anyway.

Response: We thank the reviewer for providing such an essential background on the spectrum of different antibiotics. We chose pip-tazo and ciprofloxacin in this study because those are the two antibiotics often used in many hospitals in Toronto (we had the same setting in our previous study with differential equation models: doi.org/10.1371/journal.pone.0171218). We believe such an assumption may lead to an answer to the practical situation. However, we agree with the reviewer that the trade-off may be different if we pick two drugs without cross-resistance. We choose not to present too many results in a single study and will modify our model to investigate the other case.

MINOR COMMENTS

I have some suggestions regarding readability. The authors illustrate their model by using real bug and drug names (pseudomonas, pipe-tazo etc) which can be helpful to a clinical readership, however this is quickly lost in the figures and tables where strains and drugs are designated by letters and numbers. The authors might consider using explicit (or even standard) acronyms for bugs (Pa and non-Pa) and drugs (TZP, CIP, IPM and so on, see https://journals.asm.org/journal/aac/abbreviations for widely-used acronyms) in tables, figures and throughout the text.

Response: We have adopted the suggestion from the reviewer in the revised version.

Figures 4, 5, 7. The use of one-dimensional heatmaps hampers readability in my opinion, especially if each panel's color ramp is on a different scale depending on the outcome. This forces the reader to distinguish subtle tonal changes and to read the time from top to bottom which is counterintuitive. All these panels represent time-dependent variations of outcome and might be clearly represented as line charts with time on the X-axis, outcome on th eY-axis and an horizontal line showing the baseline value. Doing this would make explanations such as "Heat maps colored in blue and red mean that de-escalation could be either beneficial or harmful under the corresponding measurement subject to the observation timing." unnecessary. For the most important outcomes, using line charts would also allow to add confidence bands to let the reader appreciate the variability of the outcome across the model simulations.

Response: We thank the reviewer for the helpful suggestions. We have completely revised the presentation of our results in the updated version.

Reviewer #3*: This paper presents a novel agent based model formalizing transmission of susceptible and resistant bacteria between patients and HCWs in an ICU under different antibiotic strategies. Specifically, the authors explore here the impact of de-escalation, by opposition to continuation.*
The authors then use the model and simulations to evaluate to which extent evaluating the compared benefit of the two strategies would be possible from randomized control trials.
The question addressed here is original and of great scientific interest. The model is well described. However, I don't believe the results presented here provide a clear and general answer to the addressed questions. Parameters choices are poorly discussed. Stochasticity is not explored and no sensitivity analysis is carried out on key assumptions such as the ward size or ward organization (number of HCWs per patient for example, mixing across groups), testing delay etc.

Response: In the new submission, we have significantly revised our illustrations on parameter selection and calibration, re-designed and presented all of our figures, and conducted additional experiments to perform sensitivity analysis on the ward size. We hope the revised version will help clarify the questions raised by the reviewer.

Major comments:

- *Because the study was specifically applied to P aeruginosa, I think the bacteria under study (P aeruginosa) should be specified in the title or at minimum in the abstract*

Response: We have specified *P aeruginosa* in the abstract of the revised manuscript.

- *Size of ward – the assumed size is 16 beds (rather small), with 4 patients per HCW shift. If I understand well, that means that there is no mixing between subgroups of patients - how realistic is it in the ICU? What would be the effect of relaxing that strong hypothesis on the results?*

Response: Yes, we assume that the pathogens are only transmitted from HCWs to patients. According to CDC (https://www.cdc.gov/hai/infectiontypes.html), healthcare-associated infections (HAIs) include central line-associated bloodstream infections, catheter-associated urinary tract infections, and ventilator-associated pneumonia, which are all majorly caused by the transmission of pathogens from invasive devices and procedures to treat patients. Therefore, most HAIs are transmitted from HCWs to patients via treatments, and patient-to-patient transmission is considered rare in this study.

What's the level of stochasticity in the model?

Response: Firstly, all individual events (death, discharge, infection, and transmission) are random events. Secondly, our main conclusions on each scenario are drawn on the statistical analysis of 1,000 simulations with randomly perturbed parameter values for each simulation – which mimic the performance of a large number of clinical studies in ICUs with close environmental conditions. Thus the stochasticity lies in both random model events and stochastic environmental conditions.

Most results show average outcomes over 500 simulations and 95% confidence interval of the mean (which strongly depend on the number of simulations) although stochasticity might strongly depend on population size. Are some extinctions observed?

Response: We presume the results mentioned by the reviewer refer to what we had in figure 3 of the original submission (now Figure 4 in the new submission). We present all results with 1,000 simulations for each experiment in the new version. We also compare the corresponding confidence and mean with experiments with more simulations and found that the results from 1,000 simulations can sufficiently represent the distribution of the outcome measurements.

We have included several individual trajectories in Figure 4 to show the randomness of each experiment. Extinctions of pseudomonas strains have been observed for some periods of time. Still, since we assume patients could be admitted with Pseudomonas strains, the extinctions do not last forever in any case.

- *Instead of evaluating the number of ICUs (100) in the study, what if population sizes were bigger in each ICU? A review of clinical trials is missing indicating classic number of arms and wards' sizes as a comparison.*

Response: We thank the reviewer for the excellent suggestion on the ICU size. The revised version has incorporated more experiments investigating ICUs with different sizes while maintaining a fixed patient-HCW ratio. We found that our conclusions do not vary according to ICU sizes. In addition, we reviewed the number of arms in the prior clinical trials and compared that with our findings in the new submission. We also conducted a brief search on the wards' sizes in the prior clinical trials but did not find clear records - as the ICU size varies significantly from hospital to hospital.

- *Illustrations of one or two typical simulated trajectories (not means) are missing and would help illustrate and understand how pathogens cohabit and evolve over time under antibiotic treatment over time.*

Response: We thank the reviewer for this helpful suggestion. In the revised version, we present several simulated trajectories together with the means in Figure 4 and provide corresponding discussions.

- *If I understand well, antibiotics are only prescribed to individuals presenting an infection here. I think antibiotic exposure is very common in ICUs for many reasons. Are the resulting % of exposure assumed here realistic in this context? Could antibiotic exposure be shown on a figure (eg additional panel in figure 3) to make it clearer to the reader?*

Response: The reviewer's understanding was correct, and we assume a well-implemented antimicrobial stewardship program in the ICUs where patients only receive antibiotic treatments whenever necessary. In reality, the actual percentage of antibiotic exposure would vary from hospital to hospital and in different periods. Unfortunately, we could not get an estimation from the same data source we used for parameter calibrations (*i.e.* our collaborators from Toronto teaching hospitals).

The results presented in figure 3 (in our first submission and now Figure 4 in the new version) were the daily infected patients according to their dominant strains, which are comparable to the prevalence of resistance against cipro and pip-tazo. Our model parameters (the transmission probabilities) are calibrated so that all simulation outcomes (1,000 simulations for each parameter combination) meet the realistic measurements of these resistance prevalence data. Therefore, all trajectories shown in Figure 4 are bounded in the realistic ranges based on our parameter calibrations. And we decided not to mark the corresponding ranges in the current version.

- *Methods: because incidence is a key outcome in epidemiology, I suggest to replace the "incidencexx" terms by "model events xx"*

Response: We appreciate the helpful suggestion from the reviewer, and we have changed "incidence" to "model events" in the new submission.

- *Are the results maintained when testing delay is shorter?*

Response: Samples collected from infected patients are submitted for antimicrobial susceptibility testing (AST), with the typical testing delay being 3 days (https://www.cdc.gov/drugresistance/ar-lab-networks/domestic/expanded-ast.html#:~:text=AST%20is%20a%20type%20of,the%20development%20of%20antibiotic%20resistance). Based on our prior communication with collaborators from Toronto teaching hospitals (where we collected other data for calibration purpose), the 3-day testing delay is typical and unlikely to be shortened. Rapid diagnosis requires the development of revolutionary techniques and is not currently available in most hospitals. Therefore, we choose not to perform a sensitivity analysis on the testing delay in the current study. Instead, we provide a brief discussion addressing our model's applicability on such a topic.

- *Parameters and Calibration paragraph: the references listed in Table 1 to justify fixed parameters values should also be included in the main text.*

Response: We have updated the references in the parameter table and revised our paragraph on the parameters and calibration in the new version. Most of the model parameters are selected based on our previous deterministic model about the same topic (doi.org/10.1371/journal.pone.0171218) and studies of our collaborators from Toronto teaching hospitals (doi: 10.1016/j.epidem.2012.12.001). We refer to the thorough discussions on the parameter estimations in the studies mentioned above. If the reviewer thinks it is necessary, we are willing to include a detailed discussion in a further revised version.

- *Figure 4: scales and labels of x- and y-axes are unclear and should be specified. More generally, there are too many figures, not easily readable and interpretable for the reader. An effort should be made to highlight the most significant results and summarize them into a selection of figures.*

Response: We agree with the reviewer about the comments on our figures. We realized that the presentation of our results in terms of figures in the previous version posed severe problems for readers in understanding the main results. So we made a considerable effort to enhance the presentations of all figures in the new version. And we would be willing to make further changes wherever the reviewer sees fit.

- *The formula used to determine sample size should be provided somewhere (either in the text or supplement)*

Response: We have provided the formula in our publicly shared code in the new version.

----

*Have the authors made all data and (if applicable) computational code underlying the findings in their manuscript fully available?
The PLOS Data policy requires authors to make all data and code underlying the findings described in their manuscript fully available without restriction, with rare exception (please refer to the Data Availability Statement in the manuscript PDF file). The data and code should be provided as part of the manuscript or its supporting information, or deposited to a public repository. For example, in addition to summary statistics, the data points behind means, medians and variance measures should be available. If there are restrictions on publicly sharing data or code —e.g. participant privacy or use of data from a third party—those must be specified.*

Reviewer #1: No: I did not have access to the code during review (the authors stated it would be available upon publication).

Reviewer #2: No: Access to software code would have been helpful to assess the code's useability.

Reviewer #3: No: but they mention somewhere in the text that this will be made available upon acceptance

Response: We have included a publicly accessible link of the main codes of our model and provided the link in our new manuscript.
