# Supplementary material for: An agent-based model on antimicrobial de-escalation in intensive care units: Implications on clinical trial design
Source: PLoS One. 2024 Apr 16;19(4):e0301944. doi: 10.1371/journal.pone.0301944 (PMC11020418; doi:10.1371/journal.pone.0301944)
Supplement: Attachment — Submitted filename: response to reviewers.pdf [file pone.0301944.s003.pdf]

Response to Reviewer's Comments

Reviewer #1: 1. The P. aeruginosa may be not the major pathogen healthcare associated infections at present status in most hospitals, including America, Asia, and Europe regions.

Response to reviewer: *P. aeruginosa* is among the five principal nosocomial pathogens as identified by World Health Organization (WHO), is listed as CDC's antibiotic resistance serious threats in the United States and is regarded as a pathogen with critical priority (priority 1) for research and development of new antibiotics. Additionally, we focus on *P. aeruginosa* in this study because its infections usually occur in people staying in the hospitals, it acquires intrinsic resistance at a high rate, and drug-resistant *P. aeruginosa* cannot be treated by nearly all broad-spectrum antibiotics.

Report on the Endemic Burden of Healthcare-Associated Infection Worldwide. World Health Organization; Geneva, Switzerland: 2011.
2019 AR Threats Report: Antibiotic Resistance Threats in the United States. Centers for Disease Control and Prevention.
Global Priority List of Antibiotic-Resistant Bacteria to Guide Research, Discovery, and Development of New Antibiotics. World Health Organization; Geneva, Switzerland: 2017.

2. The drug resistance of Pseudomonas spp. had several factors, including water and environment contaminations.
3. Needs more factors to confirm this hypothesis.

Response to reviewer: We thank the reviewer for pointing out other factors such as water and environment contaminations as other important ways for *Pseudomonas* to acquire resistance. A further dive in the literature has truly enabled us to learn more. Based on our understanding, patients colonized/infected by resistant *Pseudomonas* strains may shed bacteria in the environment which makes the environmental bacteria load fluctuating depending on the prevalence of *Pseudomonas* among patients. Incorporating such dynamics in the current model will pose further challenges in parameter estimations and we do not know how the outcomes may look like. We think considering these factors would improve our understanding and doing so may require as much efforts as starting a new project. Therefore, we discuss the limitation of this study and regard the reviewer's suggestion as the future direction in the revised manuscript. Furthermore, we would like to point out that the current model parameters, r_1 and r_2 (which relate to the probability for a newly admitted patient carrying drug resistant *Pseudomonas* strains), indirectly reflect the environmental factors of *Pseudomonas* contamination. Our codes are made available online in a reader-friendly version, and we believe readers interested in the model can easily modify the parameters to obtain answers to their own questions.
