# Supplementary material for: An agent-based model on antimicrobial de-escalation in intensive care units: Implications on clinical trial design
Source: PLoS One. 2024 Apr 16;19(4):e0301944. doi: 10.1371/journal.pone.0301944 (PMC11020418; doi:10.1371/journal.pone.0301944)
Supplement: Attachment — Submitted filename: Response to Reviewers_second round final.pdf [file pone.0301944.s004.pdf]

Response to Reviewers

Reviewer #1:

1. *P2 line 28-30, de-escalation of antibiotics focuses on susceptibility of pathogens (P. aeruginosa in this manuscript). The dual- drug resistance risk is much less than empirical therapy.*

   Response: we understand the reviewer's question: since the definitive therapy is based on susceptibility profile of pathogens, then the dual-drug resistance risk in the definitive therapy stage is much less than its risk in the empirical therapy stage. However, we would like to emphasize that our main research question is to compare de-escalation vs. continuation, which are two drug use strategies (both involve empirical stage and definitive stage) only differed in their definitive therapy stages. And what we tried to express in the sentence mentioned is that it is intuitively uneasy to envision the benefit of de-escalation on the population-level prevalence of the dual-drug resistance. This is because de-escalation can lead to frequent use of both narrow-spectrum and broad-spectrum drugs, exposing the patient population to both drugs, and the selection pressure could trigger more spontaneous mutation thus result in higher dual-drug resistance prevalence.

2. *P3, line 87-89, ciprofloxacin (fluoroquinolones antibiotics) has high resistant rate in P. aeruginosa. Why do not consider ceftazidime as the de-scalation choice?*
   Response: we consider ciprofloxacin because it is the suggested drug for de-escalation used in Toronto teaching hospitals by their antimicrobial stewardship program. And in our prior collaboration with the physicians in Toronto (doi.org/10.1371/journal.pone.0171218), we used differential equation models to study the impacts of de-escalation under the same assumption. Further, comparing to ceftazidime (which is used to treat many infections such as bladder, bone, joint, *etc.*), ciprofloxacin is usually prescribed to treat an even wider variety of infections hence is used more often in ICUs. Further, the idea of de-escalation is to use antibiotics that have established high resistance prevalence whenever possible so that we can reduce the use of the most effective ones (such as pip-tazo in our model). Then the high resistant rate of ciprofloxacin qualifies it becoming a de-escalated option. In reality, there could be more than one de-escalated drug and different physicians may identify different de-escalation options. And considering multiple de-escalated drugs would be a great future direction of this study based on more realistic assumptions. We have added this explanation in the revised version.

   We greatly appreciate the reviewer spending time reading our manuscript, as well as the helpful comments.
